# How Sensitive Is the Neophallus? Postphalloplasty Experienced and Objective Sensitivity in Transmasculine Persons

**DOI:** 10.1016/j.esxm.2021.100413

**Published:** 2021-08-20

**Authors:** Lian Elfering, Tim C. van de Grift, Muhammed Al-Tamimi, Floyd W. Timmermans, Kristin B. de Haseth, Garry L.S. Pigot, Birgit I. Lissenberg-Witte, Mark-Bram Bouman, Margriet G. Mullender

**Affiliations:** 1 Department of Plastic, Reconstructive and Hand Surgery, Amsterdam UMC, Amsterdam, The Netherlands; 2 Amsterdam Public Health Research Institute, Amsterdam UMC, Amsterdam, The Netherlands; 3 Department of Medical Psychology, Center of Expertise on Gender Dysphoria, Amsterdam UMC, Amsterdam, The Netherlands; 4 Department of Urology, Amsterdam University Medical Center, Amsterdam, The Netherlands; 5 Department of Epidemiology and Data Science, Amsterdam UMC, Amsterdam, The Netherlands

**Keywords:** Gender Affirming Surgery, Phalloplasty, Sensitivity, Erotic Feeling, Sexual Function, Transgender

## Abstract

**Introduction:**

Tactile and erogenous sensitivity of the neophallus after phalloplasty is assumed to affect the sexual well-being of transmasculine persons and, ultimately, their quality of life. The experienced and objective sensation of the neophallus and their association are largely unknown.

**Aim:**

This study evaluated experienced tactile and erotic sensation of the neophallus in transmasculine persons and investigated how this was related to objective tactile sensitivity.

**Methods:**

Between August 2017 and January 2020, 59 transmasculine persons who underwent phalloplasty were recruited to participate in a prospective follow-up study. Tactile sensitivity of the neophallus and donor-site was measured (Semmes-Weinstein Monofilament test) and compared, and participants were asked to fill out a questionnaire about experienced sensation of the neophallus and sexual wellbeing.

**Main outcome measures:**

Experienced and objective sensation of the neophallus were measured by using a questionnaire and Semmes-Weinstein Monofilament scores.

**Results:**

Neophallic tactile sensitivity was significantly reduced compared to the donor-site (n = 44), with the proximal part being more sensitive than the distal part (median follow-up of 1.8 years, range 1.0–7.2)). Sensitivity of the neophallus was not significantly associated with the surgical flap used, yet increased significantly with follow-up time. The questionnaire was completed by 26 participants of which 24 (92.3%) experienced (some degree of) tactile sensitivity in their neophallus. Erogenous sensation was experienced by 23 (88.5%). Experienced and objectified tactile sensitivity were not significantly correlated (Spearmans's rho = 0.23, *P* = .26). Answers to open-ended questions showed that results often do not match expectations.

**Conclusion:**

Tactile sensation of the neophallus was reduced in most transmasculine persons and improved slowly over time. A significant association between subjective and objective measures could not be detected. Although experienced sensitivity varied between individuals, the vast majority reported to have tactile and erotic sensitivity in the neophallus.Transmasculine persons should be informed that sensitivity of the neophallus will likely be reduced.

**Elfering L, van de Grift TC, Al-Tamimi M, et al. How Sensitive Is the Neophallus? Postphalloplasty Experienced and Objective Sensitivity in Transmasculine Persons. Sex Med 2021;9:100413.**

## INTRODUCTION

Transmasculine persons may wish to undergo phalloplasty as part of their gender affirmating treatment. In general, genital gender affirming surgery has shown to improve the quality of life of transmasculine intdividuals.^1^^,^^2^ Sexual wellbeing is an important element of quality of life, which depends in part on sexual function (eg, sexual arousal, sensation and orgasm).^2^ On average, transmasculine persons report a good quality of life (QoL) after phalloplasty, however, satisfaction with sexual function falls short of the overall satisfaction after phalloplasty.^2^, ^3^, ^4^ Phallic sensation may be an important factor in experienced sexual function and wellbeeing.^1^^,^^3^^,^^5^^,^^6^ While erogenous sensation is important for sexual stimulation and arousal,^6^, ^7^, ^8^, ^9^, ^10^ tactile sensitivity may play a positive role in the psychological acceptance of the phallus as part of one's body.^6^ It is well-known that tactile sensation after phalloplasty is diminished, while erogenous sensation is altered due to embedding of the clitoris. Presently, it is largely unknown how individuals experience sensitivity after phalloplasty, how sensitivity recovers, and how objective sensitivity relates to experienced tactile and erotic sensation.

Objective measurements of neophallic sensation have been performed for multiple surgical approaches. But the evaluation of patient-reported outcomes (PROs) to assess patient perceptions, experiences, and QoL is just as important. Use of patient-reported outcome measures (PROMs) allows for more patient-centered evaluation of treatment efficacy. However, data regarding PROs on sensation of the phallus after phalloplasty are limited and PROMs validated for the transgender population are lacking.^11^ Kuenzlen et al performed a short oral survey on erogenous zones and methods to achieve orgasm, but did not compare these results with objectively measured genital sensitivity.^12^

Given the importance of postphalloplasty sensation and the lack of knowledge on how transmasculine persons experience their genital sensitivity, the aim of this study was to analyze how transmasculine persons experience their genital sensitivity after phalloplasty and how their subjective experiences (tactile and erogenous sensitivity) were related to objective sensory recovery. Based on the limited existing evidence, we expect that tactile sensation of the neophallus is decreased and that this is reflected in a reduced experienced sensitivity.

## MATERIALS AND METHODS

### Study Design and Participants

A prospective follow-up study was conducted in the Amsterdam University Medical Center (location VU medical center) between August 2017 and January 2020. Transmasculine persons who visited the outpatient clinic and had previously undergone phalloplasty (from March 2011), or were planned to undergo phalloplasty before August 2018 were eligible to participate. If informed consent was given, participants were followed up at their regular appointments to the outpatient clinic. This study was performed in accordance with 1964 Helsinki declaration and guidelines for Good Clinical Practice and was approved by our institutional medical ethical committee (no. 2017.417). All participants provided written informed consent.

### Procedures

#### Study Procedures

Eligible candidates were invited to partake in the study when they visited the outpatient clinic for regular (pre- or postoperative) consultations. If informed consent was given, they were included. In participants who had not yet undergone phalloplasty, tactile sensitivity of the planned neophallic donor-site was measured preoperatively using the Semmes-Weinstein monofilaments (SWM) test. In case of postoperative inclusion, the contralateral donor-site was measured by SWM. Follow-up measurements were performed at least 1 year after phalloplasty during standard follow-up consultations, and, if possible, repeated over time if a participant received additional consultation(s) throughout the years. The interval between the repeated SWM test was at least one year. Postoperatively, when the tactile sensitivity of the neophallus was measured, native Dutch speaking participants were asked to, voluntarily, fill out a questionnaire pertaining to sensitivity outcomes (see Outcomes). If participants had undergone an additional operation and/or reoperation of the neophallus, measurements were performed after a recovery period of at least 3 months. Measures were taken by trained research staff. Participant demographics and surgical characteristics were retrieved retrospectively from patient files and recorded on standardized case report forms.

#### Surgical Procedures

Eligibility criteria for undergoing phalloplasty conform the Dutch standard were: age ≥ 18 years, body mass index (BMI) between 18 and 30 kg/m^2^ and not smoking. Participants received medical treatments in accordance with the World Professional Association for Transgender Health (WPATH) Standards of Care,^13^ as well as extensive preoperative psychosexual counseling.^1^^,^^3^

Phalloplasty reconstructions with and without urethral lengthening were performed. Flaps used for reconstruction included the FRFF (always with urethral lengthening), ALT, superficial circumflex iliac artery perforator flap (SCIP) or a double flap combining the ALT or SCIP flap for the shaft construction with a FRFF, SCIP or labial flap for urethral lengthening. At the time of flap mobilization and phallic reconstruction, the scrotoplasty with or without urethral lengthening was performed by the second surgical team. During scrotoplasty, deglovement of the clitoris, denudation of the glans, and mobilization of the clitoris and one dorsal nerve were performed. Eventual urethral lengthening was performed by tubularizing of the infundibular tissue in between the labia minora.^14^

In all cases, a sensate cutaneous nerve was included in the flap which was coaptated end-to-end to one of the dorsal clitoral nerves. The following cutaneous nerves per flap were used: in the FRFF the lateral antebrachial cutaneous nerve, in the ALT the lateral femoral cutaneous nerve and in the SCIP, the cutaneous nerve of the thoracic nerve (Th) Th11 and/or Th12. The nerves were harvested at adequate length, dissecting them as proximal as possible. In some case of the SCIP, a nerve graft was used to get adequate length. In all cases, special attention was given to tension-free coaptations. The denuded clitoris was placed inside the basis of neophallus.

### Outcomes

#### Patient-Reported Outcomes

Because of a lack of PROMs validated for the transgender population a 20-item questionnaire in Dutch was designed in collaboration with the Department of Medical Psychology of the Amsterdam UMC. The questionnaire addressed 5 aspects of experienced sexual well-being before and after phalloplasty using a 5-point Likert-scale or open-ended questions. The questionnaire included questions on sexual orientation, tactile sensitivity and erotic sensation (1 = never to 5 = always), sexual response (1 = never to 4 = very often, 5 = not applicable), and satisfaction with genitals (1 = very dissatisfied to 5 = very satisfied). Besides questions on sexual orientation, questions of the other 4 aspects are presented in Table 1. Supplement A provides a translation of the questions addressed in this study.

#### Tactile Sensitivity Test

Tactile sensitivity of the neophallus was measured using a minikit of 5 Semmes-Weinstein monofilaments with the following filament index numbers: 2.83, 3.61, 4.31, 4.56, and 6.65.^15^^,^^16^ These 5 monofilaments were categorized into 5 levels corresponding to the bending force levels of the filaments size used:^15^^,^^16^ (i) 2.83: 0–0.07 g; (ii) 3.61: 0.16–0.4 g; (iii) 4.31: 0.6–2 g; (iv) 4.56: 4–300 g; (v) ≤6.65: ≤300 g. The cutaneous pressure thresholds (ie, static one-point discrimination) were determined in 4 areas of the (contralateral) donor-site and neophallus; (i) proximal-left-lateral, (ii) proximal-right-lateral, (iii) distal-left-lateral, (iv) distal-right-lateral (Supplement B.). The test was performed as described by Bell-Krotoski et al.^15^ As intrapatient references for the sensitivity in the participant, most ideally, sensitivity of the donor-site was measured preoperatively. When the participant was recruited postoperatively the contralateral side was measured and served as intrapatient references.

### Data Analyses

For the analyses, participants were divided into 2 groups to correct for any biases; (i) participants who filled out a questionnaire and also underwent the SWM test, and (ii) participants who only underwent the SMW test. Demographics and outcomes were described using frequencies, means and standard deviation scores (SDs) for normally distributed and medians, and ranges for not normally distributed variables. To compare the SWM with questionnaire group and SWM only group to assess bias, Student t-tests, Fisher's exact test or Mann-Whitney-U tests were used as appropriate. An overall SWM score was calculated as the average score of all 4 measured phallic locations (see Supplement B.). Tactile sensitivity of the proximal part (PP) of the neophallus was taken as the average of the scores of locations 1. and 2., sensitivity of the distal part (DP) as the average of location 3. and 4.

Differences in the SWM test scores between preoperative (donor-site) and postoperative (neophallus) measure were analyzed using linear mixed models to account for repeated measures in some of the participants. The model included a fixed effect for follow-up time and a random intercept for participant. A second model included also the type of flap to create the shaft as fixed effect, to assess differences between surgical procedures. Models were built separately for the proximal and distal part.

Spearman Rho correlation was used to assess whether PRO measures were associated with objective tactile sensitivity scores. Answers to the open-ended questions were analyzed using qualitative thematic analysis. Statistical analyses were performed using SPSS 26.0 (IBM Corp, Armonk, New York, USA). *P* values <.05 were considered statistically significant.

## RESULTS

### Sample Characteristics

**Figure 1:**
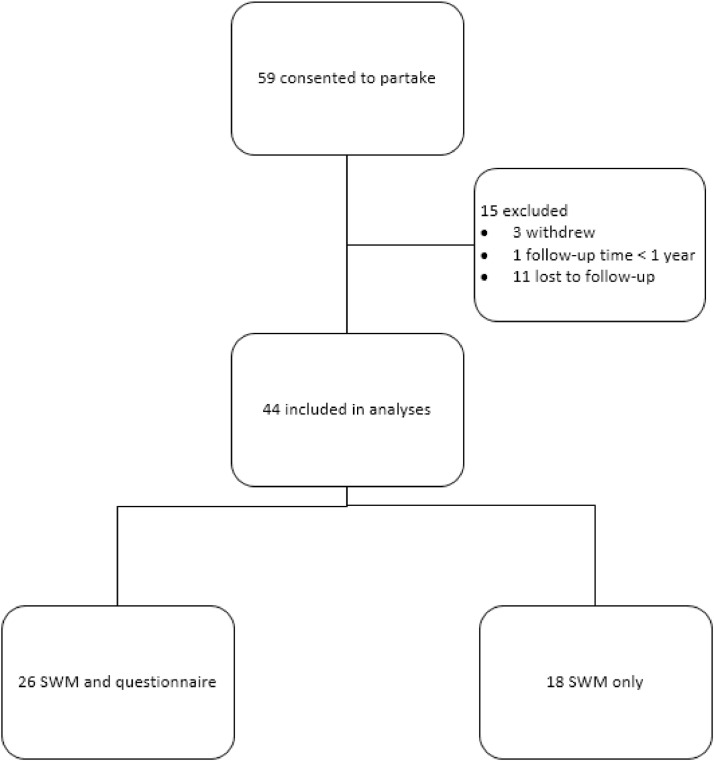
Flow chart of study participants who consented to partake in this study.

Study participation is shown in Figure 1. 59 men consented to partake in this study, of whom 15 were lost to follow-up, withdrew, or were excluded (Figure 1). In total, 44 participants were included in the analyses. All participants underwent 2 SWM tests. Either the donor-site was measured (in patients who were included preoperatively), or the contralateral donor-site was measured (in patients who were included postoperatively) as a reference value. All participants were asked during their postoperative consultation(s) to fill out the questionnaire, of whom 26 participants completed the postoperative questionnaire. During the postoperative period, 10 participants were tested twice (SWM test) over the study period of 2.5 years (total postoperative SWM-scores n = 54). Background characteristics of the participants and differences between the group of participants with “SWM only” and the group “SWM + questionnaire” are shown in Table 2. All patients were on hormone treatment during this study. The surgical technique differed significantly between the 2 groups (*P* = .016). The men, who also completed the questionnaire were treated more often with a SCIP flap (42.3% vs 16.7%), and the SWM-test only group was treated more often with a FRFF (44-4% vs 11.5%). None of the participants had medical conditions that interfered with normal wound healing or neural function. Chart review showed that 4 participants had a history of psychiatric/psychological comorbidity (eg, depression n = 2, autism n = 1, and post-traumatic stress disorder n = 1.)

**Table 1:** Outcomes sexual wellbeing questionnaire

Topic	Question	Outcome (n, %)				
Tactile feeling	Tactile sensitivity in genitals, in the last 4 wk	Never 2 (7.7)	Sometimes 1 (3.8)	Often 2 (7.7)	Very often 3 (11.5)	Always 18 (69.2)
	Location of tactile sensation	No sensation 4 (15.4)	Scrotum only 1 (3.8)	Partly in neo-phallus 1 (3.8)	Scrotum + party neo-phallus 15 (57.7)	Scrotum + total neo-phallus 5 (19.2)
Erotic feeling	Erotic feeling in neophallus, in the last 4 wk	Never 3 (11.5)	Sometimes 2 (7.7)	Often 0	Very often 6 (23.1)	Always 15 (57.7)
Sexual response		Never	Sometimes	Often	Very often	n/a
	Sexually active before phalloplasty*	7 (26.9)	6 (23.1)	6 (23.1)	5 (19.2)	2 (7.7)
	Sexually active after phalloplasty	6 (23.1)	8 (30.8)	3 (11.5)	8 (30.8)	1 (3.8)
	Able to reach orgasm through masturbation	3 (11.5)	3 (11.5)	7 (26.9)	12 (46.2)	1 (3.8)
	Able to reach orgasm through sexual activity with a partner	3 (11.5)	3 (11.5)	5 (19.2)	5 (19.2)	10 (38.5)
	Able to have penetrative sex	Yes 10 (38.5)	No 16 (61.5)			
Satisfaction with		Very dissatisfied	Dissatisfied	Neutral	Satisfied	Very satisfied
	Tactile sensitivity in genitals	1 (3.8)	6 (23.1)	5 (19.2)	8 (30.8)	6 (23.1)
	Sexual function of genitals	2 (7.7)	10 (38.5)	7 (26.9)	5 (19.2)	2 (7.7)
	Current sex life	1 (3.8)	4 (15.4)	8 (30.8)	9 (34.6)	4 (15.4)

⁎ Question answered postoperatively.

### Patient-Reported Outcomes

At follow-up, the qualitative outcomes on sexual wellbeing showed that the vast majority of participants reported to have tactile and erotic sensitivity in the neophallus (Table 1). Outcomes of the sexual wellbeing questionnaire refer to experiences in the last 4 weeks before filling out the questionnaire. Nearly everyone located their experienced erotic feelings in the area between the base of the neophallus and the scrotum. Ten participants (38.5%) were able to have penetrative sex. Experienced satisfaction with tactile sensitivity differed within the sample. The participants tended to be somewhat dissatisfied with their sexual function of their genitals however, they reported that their current sex life had improved compared with before phalloplasty.

**Table 2:** Sample characteristics participants Semmes-Weinstein monofilaments test with or without questionnaire

		Participants SWM + questionnaire *n* = 26	Participants SWM test *n* = 18	Test statistics
Mean age at phalloplasty ± SD, y		35 ± 11.8	33 ± 10.9	t (42) = -0.60, *P* = .46
Smoking (%)				
Yes		0	1 (5.6)	x^2^ (1, *N* = 44) = 1.48, *P* = .41
No		26 (100)	13 (94.4)	
Mean BMI at surgery ± SD, kg/m^2^		22.7 ± 2.2	24.2 ± 3.4	t (42) = 1.68, *P* = .11
Surgical phalloplasty techniques, (*%*)				
*Flap shaft*	*Flap urethra*			x^2^ (2, *N* = 44) = 8.40, *P* = .016
RFFF	RFFF	3 (11.5)	8 (44.4)	
ALT	n.a.	7 (26.9)	2 (11.1)	
	RFFF	4 (15.4)	5 (27.8)	
	SCIP	1 (3.8)	0	
SCIP	n.a.	4 (15.4)	2 (11.1)	
	SCIP	4 (15.4)	1 (5.6)	
	labia flap	3 (11.5)	0	
Median follow-up time after phalloplasty, y (range)				
1st measure (n = 40)		1.8 (1.0–5.3)	2.0 (1.0–4.7)	U = 203, *P* = .46
2nd measure (n = 14)		2.9 (1.2–7.2)	0	

ALT = anterolateral thigh flap; BMI = body mass index; RFFF = free radial forearm flap; SCIP = superficial circumflex iliac artery perforator flap; SD = standard deviation; SWM test = Semmes-Weinstein monofilaments test.

The responses to the open-ended question “can you explain why you are (dis)satisfied?” were categorized into 5 themes, which were then subdivided into contributing positively or negatively toward satisfaction. The most significant quotes are cited in Table 3. Factors contributing negatively were reduced tactile feeling and disappointing sexual functionality of the phallus. Not able to have penetrative sex was mentioned most frequently as a reason for not being satisfied, while some participants complained about having difficulty achieving orgasms. Some men still wanted to have an erectile prosthesis, which they expected would improve sexual function. Lastly, some responded that the appearance of the neophallus did not match their expectations, which influenced their erotic experience and self-image negatively.

**Table 3:** Significant participants’ quotes on the effects of surgery on sexual satisfaction

Theme	Contributing to dissatisfaction	Contributing to satisfaction
Tactile sensitivity	Due to less feeling in that area, touch is not always pleasant: I can hardly feel light touch.	I have sensation in the entire neo-phallus: Sensation in neo-phallus is not a requirement.
Erotic sensations	It is harder to get an orgasm. Sometimes I can't orgasm: The erotic feeling is less than before the operation: I was not aware of having a buried clitoris after surgery, which makes it more difficult to stimulate.	I'm able to get an orgasm: I'm glad the erotic feeling is still the same as before the operation.
Functionality	I can't get an erection: I can't have penetrative sex because my neo-phallus is too flexible: It does not meet my preoperative expectations.	I'm able to get an orgasm: I'm able to penetrate: I have accepted the way I am.
Aesthetics	I'm not where I want to be yet. I still want an erection prosthesis, coronaplasty and testicle prostheses. My erotic experience would then increase and it would benefit the arousal: I'm not satisfied with appearance, it's like a limp sausage.	I'm satisfied the way it is: I have accepted the way it is.
Self-image	It is not yet the way it should be; It does not meet my preoperative expectations; My neophallus is not ready yet.	Having a neo-phallus improved my self-image; The operation gave a total reduction of gender dysphoric feelings; I do love myself now; I'm the person I want to be.

On the other hand, multiple factors contributing positively to (sexual) satisfaction after phalloplasty were reported. The overall satisfaction of having a neophallus predominated in most participants. Having a neophallus improved the experienced self-image and physical confidence, as reported by many (Table 3). Furthermore, some participants commented that having received the surgery improved their sexual confidence as man and that they now dared to be sexually active with a partner.

### Tactile Sensitivity Test

**Figure 2:**
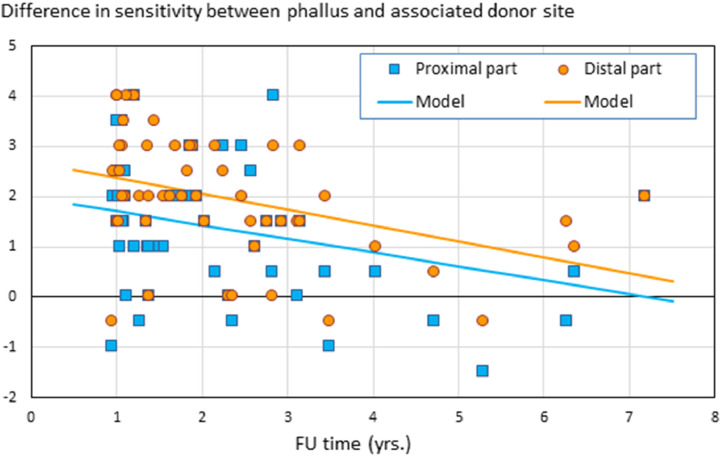
The difference in sensitivity between neophallus and associated donor-site is shown over time (n = 54). On the vertical axis the differences in the SWM test scores between preoperative (donor-site) and postoperative (neophallus) measure are shown, separately for the proximal and distal part. The lower the score, the smaller the difference in sensitivity between neophallus and associated donor-site. On the horizontal axis follow-up time in years is shown.

The SWM test was performed at various time points after phalloplasty with a median follow-up time of 1.8 years (range 1.0–7.2 years). In 44 participants, 54 SWM test outcomes were analyzed. Considerable variability in objective sensation was observed with outcomes ranging from 1 (most sensation) to 5 (least or no sensation). To account for interpatient variations in baseline sensitivity, the difference in sensitivity between neophallus and associated donor-site was taken. The sensitivity of the neophallus was significantly reduced compared to that of the donor-site, with the proximal part (PP) being more sensitive than the distal part (DP) (Figure 2, mean difference after one year follow-up 1.7 for the proximal part and 2.4 for the distal part). The surgical technique used to create the shaft was not significantly associated with SWM scores (PP: *P* = .73; DP: *P* =.052). The sensitivity increased significantly with follow-up time (PP: *P* =.016; DP: *P* =.005). However, recovery rates were slow at a rate of 0.3 points improvement per year (PP: 95%CI [-0.5, -0.05]; PP 95%CI [-0.5, -0.1]) on a scale of 1-5 (ie, “normal – no sensation”).

### Association Between Experienced and Measured Tactile Sensitivity

**Figure 3:**
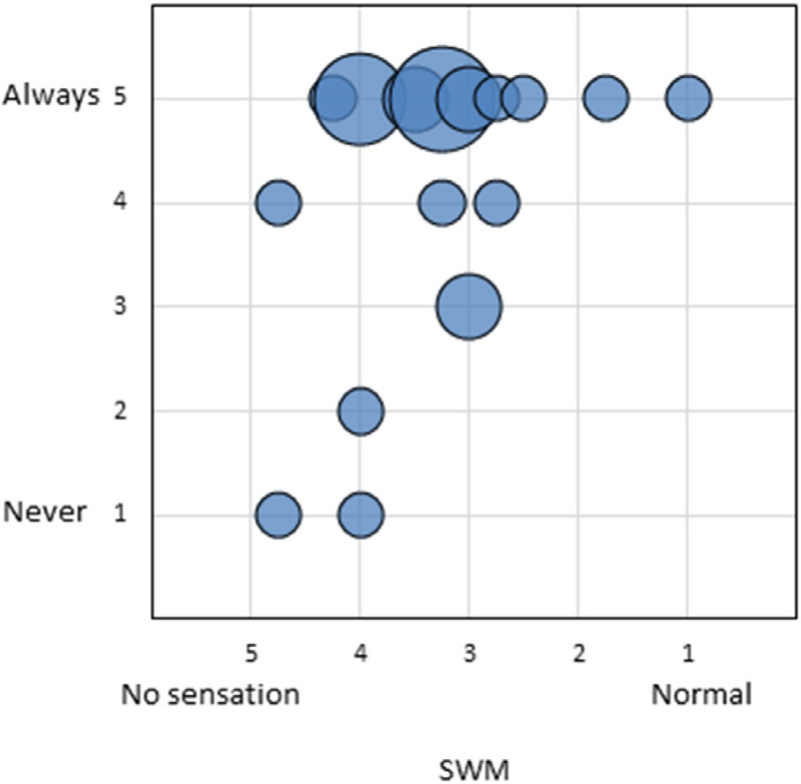
Correlation between subjective and objective tactile sensitivity (n = 54). On the vertical axis the experienced sensitivity measured by the questionnaire categorized from “never = 1” to “always = 5.” On the horizontal axis the objective sensitivity measured by the Semmes-Weinstein test categorized as “no sensation = 5” to “normal sensation = 1”.

Experienced tactile sensitivity was weakly and not significantly correlated with the tactile sensitivity as measured with the SWM test (n = 26, Spearmans's rho = 0.23, *P* =.26). Figure 3 shows that even with a low sensitivity as measured by the SWM test, multiple men reported to always have sensation in their phallus.

## DISCUSSION

Tactile sensation of the neophallus was reduced in most transmasculine persons and improved slowly over time. Remarkably, experienced and objectified tactile sensitivity were weakly and not significantly correlated, suggesting that experienced sensitivity is influenced by other factors like self-image. Objectified sensitivity of the neophallus, measured with the SWM test, was significantly reduced compared to that of the donor-site (in case of a postoperative inclusion, the contralateral donor-site). Objective sensitivity improved significantly over time, but at a slow rate indicating that sensory recovery takes multiple years and no clear end result is warranted. Furthermore, sensitivity, good functionality and improved self-image were reported by transmasculine persons to contribute to their overall sexual satisfaction.

lthough tactile and erotic sensitivity was experienced by the vast majority of participants, levels of sensation, and functionality of the neophallus varied. Patient-reported sensitivity of the neophallus has only rarely been studied previously and most of these studies focused specifically on the ability to obtain an orgasm and/or the ability to have penetrative sex.^11^^,^^12^^,^^17^ According to Kuenzlen et al, 76.4% transmasculine persons were able to obtain an orgasm after phalloplasty.^12^ In our study, most transmasculine persons could achieve an orgasm (88.5%), but the majority stated it was harder for them to achieve than before the surgery. The slightly higher rate in our study could be due to the longer follow-up time, with possibly more time for nerve regeneration, and to adapt to their altered body by developing associations with the reconstructed genitalia.

The objectified sensitivity of the neophallus, measured by the SWM test, was significantly reduced compared to that of the donor-site, with the proximal part being more sensitive than the distal part. While we found evidence that sensation improves over time, it remains unknown, when sensory recovery stabilizes and at what level. Several previous studies showed that sensory recovery of the neophallus after nerve coaptation is possible for various flap techniques, but the overall tactile sensation recovery remains suboptimal in all presented techniques.^6^^,^^8^^,^^10^^,^^12^^,^^17^^,^^18^

A limitation of this study is that various flap techniques were used. A linear mixed model (to assess the differences in the SWM test scores between preoperative and postoperative measures) was adjusted for type of flap create the shaft, whereby no significant effect was found between SWM scores., However, this study has very limited power to substantiate such differences because of the small sample size. Due to the intrinsic variability of the SWM test, and the large interindividual variation between participants, a large number of participants per group would be needed to adequately investigate the effect of surgical techniques on sensitivity outcomes. Such large group sizes are currently unavailable worldwide, such that no clear evidence exists for obvious differences between the various flap types with regard to sensitivity outcomes.

Many different surgical techniques have been described in literature for male genital Gender-Affirming Surgery (gGAS).^19^, ^20^, ^21^, ^22^, ^23^, ^24^, ^25^ Possible factors influencing sensory function after phalloplasty include: the inclusion of a sensate cutaneous nerve in the flap, whether or not this nerve is coaptated, and the success of nerve coaptation.^26^ A literature review by Morrison et al, showed that sensory preservation of the neophallus after nerve coaptation is possible. Pooled event rates suggest that some recovered glans sensitivity occurs in more than 90% of transmasculine persons, whereas erogenous sensation was present in more than 95%.^6^ With regard to technique-specific outcomes, it has been reported that the radial forearm free flap (FRFF) technique, regarded as the gold standard, often results in suboptimal outcomes regarding tactile sensation.^8^^,^^10^^,^^17^ However, Kuenzlen et al reported that the majority of the transmasculine persons showed some degree of sensitivity in the FRFF neophallus and were able to achieve orgasm by stimulating their neophallus.^12^ Holzbach et al described the use of a sensate pedicled anterolateral thigh flap (ALT) for phalloplasty, which was reported to yield some degree of sensitivity, with an ongoing reinnervation at follow-up.^18^

Nerve coaptation in FRFF phalloplasty is mostly performed by coaptation of the medial and lateral antebrachial cutaneous nerves, to one of the 2 dorsal clitoral nerves and to the ilio-inguinalis nerve or genito-femoralis nerve.^12^^,^^17^ When using this technique, patients in our center reported to experience an inconveniently sensitive neourethra. Based on these patient-reported outcomes, we have (previous to this study) ceased to connect the medial antebrachial cutaneous nerve in favor of patient comfort. Possible this may also affect the tactile sensitivity outcomes, nevertheless, our patients seem to experience the same level of tactile sensitivity as reported by Selvaggi et al and Kuenzlen et al. ^12^^,^^17^

The measured tactile sensitivity correlated only weakly with the ability to feel touch, as reported by the transmasculine persons themselves. This lack of association could be partly explained by the way by which sensitivity was measured. Participants reported whether they could feel touch in their phallus (1 = never to 5 = always), while the SWM test measures the pressure threshold to feel touch. Apparently, these measures are not interchangeable. Furthermore, experienced sensation may also be influenced by “learning.” With use of the neophallus the brain may adapt to changed peripheral stimuli, and this is known to be highly dependent on pattern and frequency of use.^27^ Similar mechanisms may affect the experience of erogenous sensitivity. Overgoor et al reported earlier on the effects of nerve repair of the penis on sexual health in cisgender men.^27^ They showed that visualization and motivation play a major role in the development of tactile and erogenous sensations. Hence, restoration of genital sensitivity could well be subject for postoperative rehabilitation training. Furthermore, neuroimaging studies observed that transgender individuals more frequently experience some degree of dissociation of bodily emotions from body representation,^28^ possibly resulting in discrepancies in bodily sensations and perceived experiences of the body.

Experienced overall satisfaction after phalloplasty varied. Several participants reported to be very satisfied with their neophallus, mostly because having a neophallus improved their experienced self-image and physical confidence. Some stated that they accepted their neophallus as it is (despite imperfections), but expressed that outcomes of phalloplasty are suboptimal, specifically referring to the reduced tactile sensitivity, long recovery time, and uncertainty about the final result. To prevent this discrepancy between expectations and outcomes it is necessary to optimize preoperative counseling. Transmasculine persons should be informed that there is a chance that postoperative sexual functionality of the neophallus will be suboptimal and that sensitivity will likely be reduced, with a long and slow recovery toward an end result that remains unclear. Also, more specialized rehabilitation after surgery is desirable as this could help the brain adapt to the changed peripheral stimuli and transmasculine persons to adapt to their changed body.

This study is of added value since it is one of few reports on experienced and measured tactile sensitivity of the neophallus. However, the study was limited by the cross-sectional design, allowing only for associative findings, and its overall small sample size, and low number of patients per specific flap used limiting its statistical power. Objective discriminatory sensibility was evaluated by using the SWM test, while Gilbert et al demonstrated that pressure and vibratory thresholds were the most reliable and informative methods for testing penile sensitivity.^10^ Besides, tactile sensitivity is only one component of sensation and consequently does not give a comprehensive picture of sensation in the phallus. Furthermore, by lack of a good alternative a nonvalidated PRO measure was used, which was administered at variable postoperative time points. Hence, these results should be seen as indicative and interpreted with some caution. Despite these shortcomings, this study provided a more comprehensive insight into the sensitivity outcomes after phalloplasty.

## CONCLUSION

This study showed that at a median follow-up time of 1.9 years after phalloplasty, the tactile sensation of the neophallus is significantly reduced compared to the neophallic donor-site. This difference seems to decrease slowly where neophallic sensibility can improve over time. Almost all transmasculine persons reported to have sensation in their neophallus although no significant association could be detected between objectively measured tactile sensitivity and the patient reported ability to feel touch. Sensory outcomes are complex and this study only measured the most superficial components of sensory experience. Based on qualitative findings, we conclude that outcomes often do not match expectations, which causes dissatisfaction in a proportion of participants. Hence, it is necessary to inform transmasculine persons extensively on what to expect after phalloplasty and discuss the considerable probability of having to face shortcomings with regard to the esthetic and/or functional outcomes as well as a reduced sensibility and long recovery time. It is suggested that transmasculine persons may benefit from special rehabilitation training to improve penile sensation and awareness, and development of postoperative rehabilitation training programs may be subject of future research.

## STATEMENT OF AUTHORSHIP

Conceptualization: L.E., T.G., M.A., F.T., K.H., G.P., B.L., M.B., and M.M.; Methodology: L.E., T.G., M.B., and M.M.; Formal Analysis: L.E., T.G., B.L., and M.M.; Investigation: L.E., T.G., M.B., and M.M.; Resources: L.E., M.A., and F.T.; Supervision: T.G., M.B., and M.M.; Writing – Original Draft: L.E.; Writing – Review & Editing: L.E., T.G., M.A., F.T., K.H., G.P., B.L., M.B., and M.M.

## Appendix Supplementary materials

Image, application 1

Image, application 2

## Footnotes

Supplementary material associated with this article can be found in the online version at doi:10.1016/j.esxm.2021.100413.

## References

[1] Grift van de TC, Pigot GLS, Kreukels BPC (2019). Transmen's experienced sexuality and genital gender-affirming surgery: findings from a clinical follow-up Study. J Sex Marital Ther.

[2] Wierckx K, Caenegem van E, Elaut E (2011). Quality of life and sexual health after sex reassignment surgery in transsexual men. J Sex Med.

[3] Grift van de TC, Pigot GLS, Boudhan S (2017). A longitudinal study of motivations before and psychosexual outcomes after genital gender-confirming surgery in transmen. J Sex Med.

[4] Rooij de FPW, Grift van de TC, Veerman H (2021). Patient-reported outcomes after genital gender-affirming surgery with versus without urethral lengthening in transgender men. J sex Med.

[5] Hage JJ, Bout CA, Bloem JJ (1993). Phalloplasty in female-to-male- transsexuals: what do our patients ask for?. Ann Plast Surg.

[6] Morrison SD, Massie JP, Dellon AL. (2018). Genital sensibility in the neophallys: getting a sense of the current literature and techniques. J Reconstr Microsurg.

[7] Gilbert DA, Horton CE, Terzis JK (1987). New concept in phallic reconstruction. Ann Plast Surg.

[8] Hage JJ, Bouman FG, de Graaf EH (1993). Construction of the neophallus in female-to-male transsexuals: the Amsterdam experience. J Urol.

[9] Hage JJ, Graaf de FH. (1993). Addressing the ideal requirements by free flap phalloplasty: some reflections on refinements of technique. Microsurgery.

[10] Gilbert DA, Williams MW, Horton CE (1988). C.J. Phallic reinnervation via the pudendal nerve. J Urol.

[11] Andreasson M, Georgas K, Elander A (2018). Patient-reported outcome measures used in gender confirmation surgery: an overview. Plast Reconstr Surg.

[12] Kuenzlen L, Nasim S, van Neerven S (2020). Multimodal evaluation of funtional nerve regeneration in transgender individuals after phalloplasty with a free radial forearm flap. J Sex Med.

[13] Coleman E, Bockting W, Botzer M (2012). Standards of care for the health of transsexual, transgender, and gender-nonconforming people, version 7. Int J Transgend.

[14] Al-Tamimi M, Pigot GLS, Sluis van der WB (2018). Colpectomy significantly reduces the risk of urethral fistula formation after urethral lengthening in transgender men undergoing genital gender affirming surgery. J Urol.

[15] Bell-Krotoski JA, Weinstein S, Weinstein C. (1993). Testing sensibility, including touch-pressure, two-point discrimination, point localization, and vibration. J Hand Ther.

[16] Weinstein S. (1993). Fifty years of somatosensory research: from the Semmes-Weinstein monofilaments to the Weinstein Enhanced Sensory Test. J Hand Ther.

[17] Selvaggi G, Monstrey S, Ceulemans P (2007). Genital sensitivity after sex reassignment surgery in transsexual patients. Ann Plast Surg.

[18] Holzbach T, Giunta RE, Machens HG (2011). Phalloplasty with pedicled anterolateral thigh flap. Handchir Mikrochir Plast Chir.

[19] Djordjevic ML, Bumbasirevic MZ, Vukovic PM (2006). Musculocutaneous latissimus dorsi free transfer flap for total phalloplasty in children. J Pediatr Urol.

[20] Felici N, Felici A. (2006). A new phalloplasty technique: the free anterolateral thigh flap phalloplasty. J Plast Reconstr Aesthet Surg.

[21] Garaffa G, Christopher NA, Ralph DJ. (2010). Total phallic reconstruction in female-to-male transsexuals. Eur Urol.

[22] Hasagawa K, Namba Y, Kimata Y. (2013). Phalloplasty with an innervated island pedicled anterolateral thigh flap in a female-to-male transsexual. Acta Med Okayama.

[23] Sengezer M, Öztürk S, Deveci M (2004). Long-term follow-up of total penile reconstruction with sensate osteocutaneous free fibula flap in 18 biological male patients. Plast Reconstr Surg.

[24] Frey JD, Poudrier G, Chiodo MV (2017). An update on genital reconstruction options for the female-to-male transgender patient: a review of the literature. Plast Reconstr Surg.

[25] Sluis van der WB, Smit JM, Pigot GLS (2017). Double flap phalloplasty in transgender men: Surgical technique and outcome of pedicled anterolateral thigh flap phalloplasty combined with radial forearm free flap urethral reconstruction. Microsurgery.

[26] Griffin MF, Malahias M, Hindocha S (2014). Peripheral nerve injury: principles for repair and regeneration. Open Orthopaed J.

[27] Overgoor MLE, Jong de TPVM, Cohen-Kettenis PT (2013). Increased sexual health after restored genital sensation in male patients with spina bifida or a spinal cord injury: the TOMAX procedure. J Urol.

[28] Lin C-S, Ku H-L, Chao H-T (2014). Neural network of body representation differs between transsexuals and cissexuals. PLoS One.

