# Supplementary material for: How Sensitive Is the Neophallus? Postphalloplasty Experienced and Objective Sensitivity in Transmasculine Persons
Source: Sex Med. 2021 Aug 20;9(5):100413. doi: 10.1016/j.esxm.2021.100413 (PMC8498953; doi:10.1016/j.esxm.2021.100413)
Supplement: Supplementary file 1 [file mmc1.docx]

**A. Tactile feeling** (almost) (almost)
 never always
A.1. Have you experienced tactile sensitivity in your genitals,
during the last 4 weeks? □ □ □ □ □

A.2. Where, in your scrotum and/or penis, do you feel tactile sensation?
Select the area(s) on the image below. □ □ □ □ □


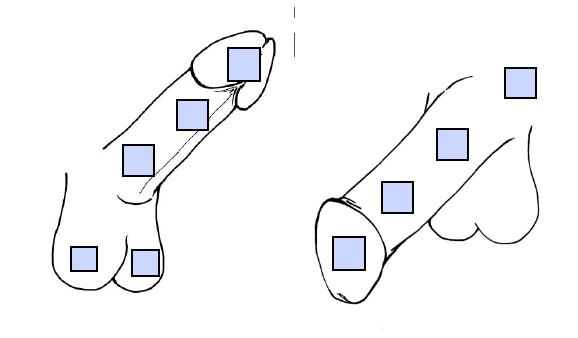


**B. Erotic feeling** (almost) (almost)
 never always
B.1. Have you experienced erogenous sensation in the neo-phallus,
during the last 4 weeks? □ □ □ □ □

B.2. When you are sexually aroused, do you experience tactile/erogenous sensation in the area of the neo-phallus? If yes, where do you feel this?

B.3. Is your erogenous sensation in your genitals changed compared to before the operation?

If yes, could you give a short comment on this?

**C. Sexual response**This section includes intimate questions. These questions address your sexual behavior. For example, these include questions on sexual activity and which sexual acts you perform.

If you do not feel comfortable to answer these questions, you can skip this section and move on to section E about satisfaction.

***Before the operation;***
C.1. Were you sexually active, in the last year before the phalloplasty operation?

Never Sometimes Often Very often N/A

□ □ □ □ □

C.2. Where you able to reach an orgasm through masturbation, in the last year before the operation?

Never Sometimes Often Very often N/A

□ □ □ □ □

C.3. Could you reach an orgasm through sexual activity with a partner, in the last year before the operation?

Never Sometimes Often Very often N/A

□ □ □ □ □

***After the operation;***
C.4. Are you sexually active since the phalloplasty operation?

Never Sometimes Often Very often N/A

□ □ □ □ □

C.5. Are you able to reach an orgasm through masturbation?

Never Sometimes Often Very often N/A

□ □ □ □ □

C.6. Are you able to reach an orgasm through sexual activity with a partner?

Never Sometimes Often Very often N/A

□ □ □ □ □

C.7. Are you able to have penetrative sex?

C.8. If yes, do you use any tools to be able to penetrate?

**D. Satisfaction**
D.1. Are you satisfied with your current sex life?

Never Sometimes Often Very often N/A

□ □ □ □ □

D. 2. Are you satisfied with the tactile sensitivity in your genitals?

Never Sometimes Often Very often N/A

□ □ □ □ □

D. 3. Are you satisfied with the sexual function of your genitals?

Never Sometimes Often Very often N/A

□ □ □ □ □

D.4. What makes you feel (dis)satisfied?

D.5. Can you shortly elaborate on whether and how the genital affirmation surgery influenced your sex life? Do you experience your sexuality differently? Is your relation with a (sexual) partner changed (if applicable)?
