# Supplementary figures and images for: How Sensitive Is the Neophallus? Postphalloplasty Experienced and Objective Sensitivity in Transmasculine Persons
Source: Sex Med. 2021 Aug 20;9(5):100413. doi: 10.1016/j.esxm.2021.100413 (PMC8498953; doi:10.1016/j.esxm.2021.100413)

**Supplement A.**

Neo-phallus

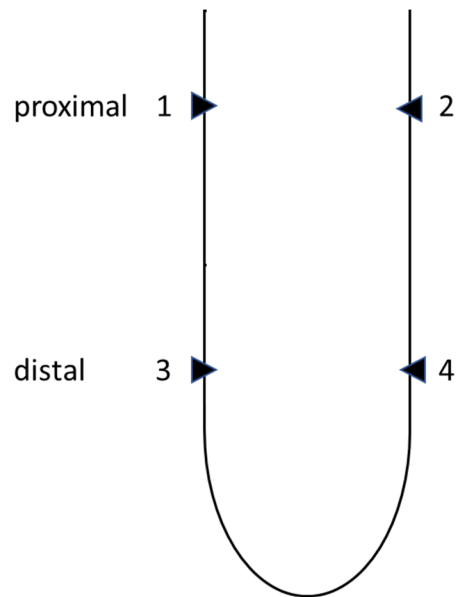

Supplement: Supplementary file 2 [file mmc2.pdf]
